# Can Semilocal Approximations to the Embedding Potential Tackle Charge-Transfer-to-Solvent Excitations? An Aqueous Thiocyanate Example

**DOI:** 10.1021/acs.jctc.5c00991

**Published:** 2025-10-06

**Authors:** Pierre-Olivier Roy, Mingxue Fu, Ronit Sarangi, Anna I. Krylov, Tomasz A. Wesolowski

**Affiliations:** † Départment de Chimie Physique, Université de Genève, Quai Ernest-Ansermet 30, CH-1211 Genève 4, Switzerland; ‡ Department of Chemistry, 2029 Syracuse University, Syracuse, New York 13244, United States; § Department of Chemistry, 5116 University of Southern California, Los Angeles, California 90089-0482, United States

## Abstract

Frozen-density embedding theory (FDET) provides a formal basis for methods employing density-dependent embedding potentials. FDET-based methods involve approximations concerning: (i) the choice of the approximant for the bifunctional *v*_xct_^nad^[ρ_A_,ρ_B_], (ii) the localization of the embedded wave function, and (iii) the approach used to generate the electron density of the environment. The set of approximations that has been shown to yield highly accurate complexation-induced shifts in vertical excitation energies for valence excitations localized on the chromophore—referred to as the “standard FDET protocol”—is expected to fail if applied to charge-transfer-to-solvent excitations. This work illustrates that such excitations can also be treated using an FDET-based method; however, they require refinement of the approximations used in the standard protocol.

## Graphical abstract

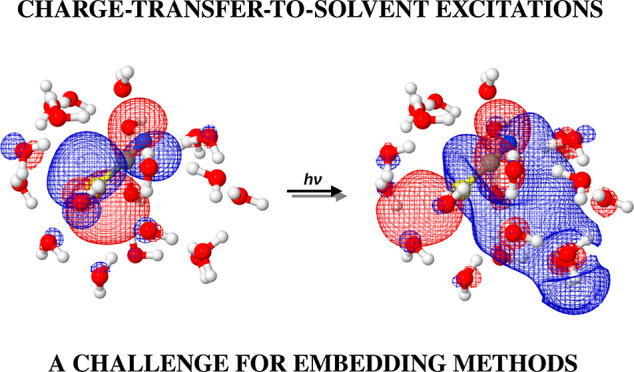

## Introduction

Solvatochromism—changes in a chromophore’s absorption and emission spectra due to interactions with the solvent—plays a crucial role in chemistry and physics. Solvatochromic effects can significantly influence spectral properties, altering energy levels, transition intensities, and charge distributions. Thus, they must be considered when modeling phenomena in environmental chemistry, optoelectronics, and biological systems.

Many computational strategies have been developed to incorporate solvent effects into quantum chemical calculations following the multiscale philosophy. Models that treat the environment either as a continuum or at atomic resolution (QM/MM) are widely used, but the usual underlying approximations limit accuracy when solute–solvent interactions are strong. Frozen-density embedding theory (FDET) provides a rigorous formal framework that encompasses both the continuum^1^ and atomistic^2^ representations of the solvent or other microscopic environments and allows for the systematic improvements of embedding models. FDET provides the exact relations between the embedding potential, embedded wave function, and the Hohenberg–Kohn energy functional. These relations were formulated for different treatments of the electron–electron interactions in the ground^3–5^ and excited states.^6,7^ The total electron density of the system is constructed as a sum of two components: (1) ρ_A_(**r**) constructed from the embedded wave function (Ψ_A_) and (2) ρ_B_(**r**), the density representing the remaining electrons. The total electronic energy of the system is then expressed as a functional depending on two independent quantities: Ψ_A_ and ρ_B_. The embedded wave function is thus an auxiliary quantity to optimize ρ_A_. In FDET-based methods, the solute can be treated with any quantumchemistry method that is adequate for a given observable, whereas the electron density ρ_B_ associated with the environment is constructed by the practitioner based on the properties of the molecules in the environment. Most common applications of FDET-based methods construct ρ_B_ using lower-cost quantum-chemical calculations (see the dedicated reviews in refs 8–10 , for instance). They can also be used in association with ρ_B_ constructed from statistical-mechanics based methods^1,11^ or even experimental data.^12^ Despite the flexibility in choosing the environment’s representation, the effect of the solvent on the embedded wave function is treated fundamentally quantum mechanically. These effects are represented in the FDET embedding potential, which incorporates both classical electrostatics as well as the fermionic nature of electrons, thus ensuring a physically consistent treatment of the key electronic effects. The nonclassical component of the embedding potential is represented by means of a bifunctional, *v*_xct_^nad^[ρ_A_,ρ_B_], which depends on the density of the embedded wave function (ρ_A_) and the density of the environment (ρ_B_) and which must be approximated in practice.

FDET provides a formal foundation for inexpensive computational methods capable of describing the effect of the environment on the electronic properties with remarkable accuracy for various types of noncovalent interactions between the embedded system and the environment. Benchmark studies on 351 electronic excitation energies using a method based on FDET applying rather simple approximations (which we refer to in this work as “standard FDET protocol”) have shown that the average absolute error due to the approximations used is 0.039 eV for local excitations in noncovalently bonded systems.^13^ The maximum error of 0.188 eV was encountered for large solvent shifts (0.907 eV). The error tends to increase as the overlap between the charge density of the embedded system and the environment increases. In the standard FDET protocol only atomic basis sets centered on the chromophore are used to represent the embedded wave function. We refer to it as the “monomer expansion approximation” (MEA). MEA is commonly used in conventional embedding methods, such as continuum models, QM/MM, polarizable force fields, effective fragment potentials, etc. It is so common that it is not even explicitly mentioned as an approximation in publications using such methods. MEA contributes to the computational efficiency of the standard FDET protocol. It is, however, not adequate for some embedding scenarios. For instance, if the environment absorbs light in the same range as the chromophore, the dynamic response of the environment is neglected.^8^ The most pronounced failure of MEA in FDET occurs for chromophore dimers, when one monomer is described by Ψ_A_ and another by ρ_B_. Such cases can be treated by embedding techniques that go beyond FDET, such as subsystem DFT—originally proposed by Cortona^14^ and later generalized to excited states by Casida and Wesolowski.^15^ The method introduced by Neugebauer^16^ uses other descriptors for the environment besides the charge density. MEA is also not adequate for transitions involving charge-transfer between the environment and the chromophore. Two cases have to be distinguished. If charge is transferred from the chromophore to the environment, MEA could handle this under two conditions: (i) the basis set used in MEA for the chromophore must include diffuse functions extending into the environment, and (ii) the approximation used for the FDET embedding potential must properly describe the embedding potential near the molecules in the environment. The case of the charge-transfer from the environment to the chromophore is outside the domain of applicability of FDET-based methods but for a different—more fundamental—reason. In FDET, the total density in the excited state is obtained as a sum of the density generated from the embedded wave function and the density representing the environment. Obtaining accurate total density from FDET thus requires a prior knowledge of the density of the molecules in the environment in the excited state of the entire system to make sure that ρ_B_(**r**) is always smaller than the total density for the excited state in the vicinity of the solvent molecules (thereby ensuring that the non-negativity condition for the target embedded density is not violated^17^).

In the standard FDET protocol, which was successfully applied in ref 13, ρ_B_ was generated without taking into account the effect on the density due to interactions between the chromophore and the environment. ρ_B_ was generated as a superposition of densities of isolated molecules of the environment. Other possibilities were considered in the literature. Khait and Hoffmann^18^ introduced an FDET-based method for excited states, in which the environment density is optimized using the freeze-and-thaw procedure.^19^ Such construction of the environment density is used in various FDET-based methods. However, the physical interpretation of such construction of ρ_B_ is not possible because the simultaneous minimization of the two components of the total density does not yield a unique pair of densities in the case of the exact *v*_*t*_^nad^[ρ_A_,ρ_B_].^8^ If approximants are used for *v*_xct_^nad^[ρ_A_,ρ_B_], the optimization of ρ_B_ reflects both the electronic polarization and the minimization of the error in the energy functional corresponding to the used *v*_xct_^nad^[ρ_A_,ρ_B_]. Another way to prepare the density of the environment, prepolarization,^20^ entails polarizing it by the electric field due to the embedded chromophore, which is physically meaningful if MEA is used. It further improves the accuracy of the transition energies and transition moments but the improvements are usually much smaller than the full effect of the environment on these quantities.^21,22^

Rydberg states^124^ and charge-transfer-to-solvent (CTTS) excitations represent a particularly challenging case for embedding because they involve diffuse states that penetrate into the solvent. This diffuse nature makes these transitions highly sensitive to changes in the structure of the solvent around the solute. Aqueous thiocyanate (SCN^–^) serves as a challenging test case due to its chaotropic nature, which disrupts the hydrogen-bond network of water, and due to its CTTS states. As illustrated in Figure 1, which shows natural transition orbitals (NTOs) for 4 electronic transitions of SCN^–^(aq), some of the states have localized wave functions while others extend into the solvent cavities, resembling the states of a solvated electron. Previous studies^23–124^ have highlighted the strong dependence of the aqueous SCN^–^ absorption spectrum on solute–solvent configurations, demonstrating the importance of an accurate description of solvent structure.

**Figure 1:**
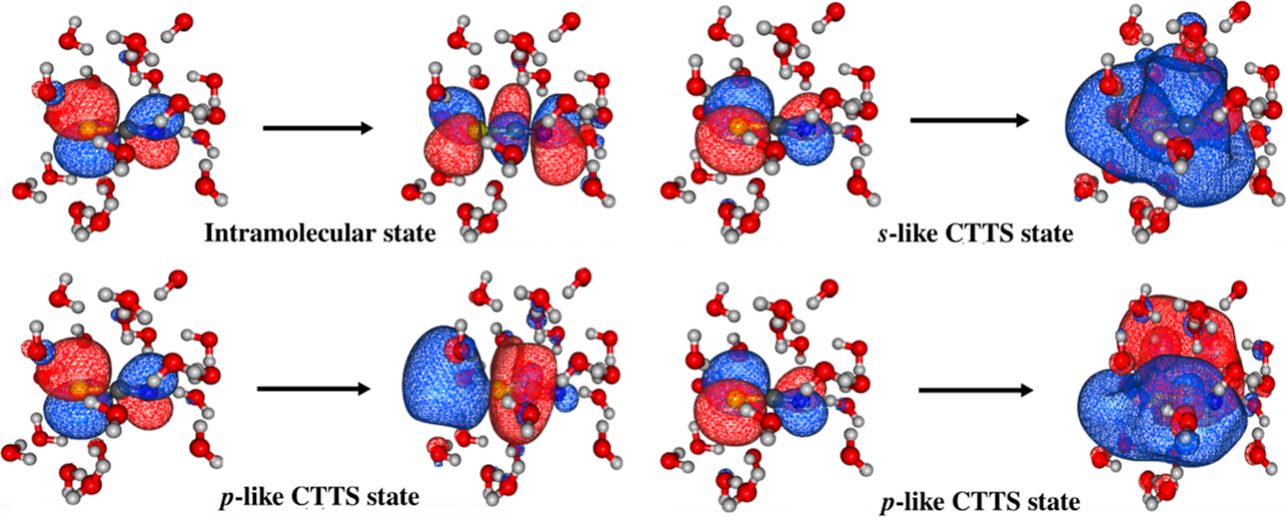
NTOs of the lowest excited states in aqueous thiocyanate. Orbital images are adapted with permission from ref 25. Available under a CC-BY-NC-ND license. Copyright 2022 Ronit Sarangi and Anna I. Krylov.

To correctly describe these states (as well as Rydberg states^124^ of the solvated molecules), a theoretical framework must strike a balance between two inherently quantum mechanical effects—stabilization by the delocalization of electronic density into the solvent and the confinement effects imposed by Pauli exclusion principle. Many embedding models struggle to provide a physically sound description of these effects, which arise from the fermionic nature of electrons and lead to spatial restrictions on electronic density. Conventional embedding schemes often rely on approximations that fail to capture these effects adequately, resulting in errors in the computed electronic structure and spectral properties. To overcome the deficiencies of simple electrostatic embedding, refs 23 and 24 employed a brute force approach and used very large embedded subsystems, consisting not only of the chromophore, but also up to ∼20 water molecules.

In principle, such cases could still be treated with FDET-based methods by extending the basis sets into the environment and thus allowing electron density redistribution between solute and solvent. However, such an approach makes the results highly susceptible to any inaccuracies in the nonadditive exchange-correlation-kinetic potential, *v*_xct_^nad^[ρ_A_,ρ_B_]. Indeed, the conventional semilocal approximations for this potential—successful for systems without significant charge-transfer excitations—fail for aqueous SCN^–^ which we will demonstrate in the first part of the result section. In the second part, we show how to overcome this failure. The use of *v*_*t*_^nad(NDCS)^[ρ_A_,ρ_B_]—the recently introduced approximant for the kinetic component of *v*_xct_^nad^[ρ_A_,ρ_B_][Bibr ref26]—improves qualitatively the accuracy of the energies and oscillator strengths for these states. Owing to the use of this approximant, which better describes the embedding potential near the nuclei in the environment than conventional decomposable approximants, the FDET results are numerically stable even if the basis sets used for the embedded wave function comprises atomic basis sets centered on the nuclei belonging to the solvent. By focusing on the prototypical CTTS excitations of aqueous thiocyanate, we subject FDET to a stringent test in which the solute and solvent densities overlap substantially. Our study aims to provide guidance on the applicability of FDET to challenging systems.

## Methods

We used the FDET-based method to compute the excitation energies and oscillator strengths of SCN^–^ embedded in small water clusters. The FDET energy functional $E_{v_{AB}}^{FDET}\left[\Psi_{A},\rho_{B}\right]$ depends on two independent variables: (1) the *N*_A_-electron embedded wave function Ψ_A_ and (2) a non-negative real function ρ_B_(**r**) integrating to an integer (*N*_B_) which we refer to as the electron density of the environment. $E_{v_{AB}}^{FDET}\left[\Psi_{A},\rho_{B}\right]$ is consistent with the Hohenberg–Kohn energy functional $E_{v_{AB}}^{HK}\left[\rho \right]$ ,^27^ i.e., it satisfies the basic FDET equality

1 $$\min_{\Psi_{A}\to N_{A}}E_{v_{AB}}^{FDET}\left[\Psi_{A},\rho_{B}\right]=E_{v_{AB}}^{FDET}\left[\Psi_{A}^{o},\rho_{B}\right]=E_{v_{AB}}^{HK}\left[\rho^{\Psi_{A}^{o}}+\rho_{B}\right]$$

where $\rho^{\Psi_{A}^{o}}\left(r\right)=\left\langle \Psi_{A}^{o}\left|\sum_{i=1}^{N_{A}}\delta \left(r_{i}-r\right)\right|\Psi_{A}^{o}\right\rangle$ and *v*_AB_(**r**) is the external potential defining the entire system.

The optimization of the embedded wave function proceeds by solving the following FDET eigenvalue problem^4^

$$\begin{array}{ll} \left({Ĥ}_{A}+{\mathrm{v̂}}_{emb}^{FDET}\left[\rho_{A}^{j},\rho_{B};v_{B}\right]\right)\Psi_{A}^{j}=\lambda_{A}^{j}\Psi_{A}^{j} & \left(2\right)\\ \rho_{A}^{j}\left(r\right)=\left\langle \Psi_{A}^{j}\left|\sum_{i=1}^{N_{A}}\delta \left(r_{i}-r\right)\right|\Psi_{A}^{j}\right\rangle & \\ v_{emb}^{FDET}\left[\rho_{A}^{j},\rho_{B};v_{B}\right]\left(r\right)=v_{B}\left(r\right)+\int \frac{\rho_{B}\left(r^{\prime }\right)}{\left|r-r^{\prime }\right|}dr^{\prime }+v_{xct}^{nad}\left[\rho_{A}^{j},\rho_{B}\right]\left(r\right) & \left(3\right) \end{array}$$

where ${Ĥ}_{A}$ is the isolated Hamiltonian corresponding to *N*_A_ electrons, $v_{xct}^{nad}\left[\rho_{A},\rho_{B}\right]\left(r\right)={\frac{\delta E_{xct}^{nad}\left[\rho ,\rho_{B}\right]}{\delta \rho \left(r\right)}|}_{\rho =\rho_{A}}$ , and *E*_xct_^nad^[ρ_A_,ρ_B_] is nonadditive exchange–correlation and kinetic energy functional determined uniquely by the pair of electron densities ρ_A_(**r**) and ρ_B_(**r**) defined in ref 4.

According to Perdew–Levy theorem,^28^ stationary solutions of eq 2 beyond the ground state can be interpreted as excited states

4 $$\begin{array}{lll} \varepsilon_{j} & = & E_{v_{AB}}^{HK}\left[\rho_{A}^{j}+\rho_{B}\right]-E_{v_{AB}}^{HK}\left[\rho_{A}^{o}+\rho_{B}\right]\\ & = & E_{v_{AB}}^{FDET}\left[\Psi_{A}^{j},\rho_{B}\right]-E_{v_{AB}}^{FDET}\left[\Psi_{A}^{o},\rho_{B}\right] \end{array}$$

The second equality in the above equation follows directly from the basic FDET equality for energy given in eq 1.

In FDET, calculations of vertical excitation energy are affected by the state-specificity problem in both ρ_A_ and ρ_B_. In the linearized FDET variant, where the functional *E*_xct_^nad^[ρ_A_,ρ_B_] is expanded around some reference density ρ_A_^ref^ (see Section Computational Details), the FDET embedding potential in eq 3 is ρ_A_-independent.^7^ In this case, the vertical excitation energy for the *j*-th excited state is simply the difference of the expectation values between the ground state and excited state wave functions, with the same ρ_B_ applied for all states

5 $$\begin{array}{lll} \varepsilon_{j} & = & E_{v_{AB}}^{FDET}\left[\Psi_{A}^{j},\rho_{B}\right]-E_{v_{AB}}^{FDET}\left[\Psi_{A}^{o},\rho_{B}\right]\\ & \approx & \langle \Psi_{A}^{j}|{Ĥ}_{A}+{\mathrm{v̂}}_{emb}^{FDET}\left[\rho_{A}^{ref},\rho_{B};v_{B}\right]|\Psi_{A}^{j}\rangle -\langle \Psi_{A}^{o}|{Ĥ}_{A}+{\mathrm{v̂}}_{emb}^{FDET}\left[\rho_{A}^{ref},\rho_{B};v_{B}\right]|\Psi_{A}^{o}\rangle \\ & = & \lambda_{A}^{j}\left[\rho_{A}^{ref},\rho_{B}\right]-\lambda_{A}^{0}\left[\rho_{A}^{ref},\rho_{B}\right] \end{array}$$

If different ρ_B_ is used for different states—polarized by the corresponding excited state of embedded chromophore for instance—the orthogonality between the embedded wave functions for different states can also be preserved if the higher than linear terms in the expansion of the Hohenberg–Kohn energy functional in ρ_A_ and ρ_B_ are neglected (see ref 22 for the exact expression).

Concerning the interpretation of other than the lowest energy solutions of the FDET eigenvalue equation (eq 2), which was derived in ref 4 for ground states, as excited states. We note that Khait and Hoffmann were the first to use this interpretation in the computational method they developed.^18^

Regarding the choice of the reference density ρ_A_^ref^ used for linearization of the *E*_xct_^nad^[ρ_A_,ρ_B_] bifunctional, our previous studies indicated the adequacy of using the ground-state density of the isolated chromophore, even for excitations involving significant delocalization of electron density within the embedded species such as in the case of hydrated uracil.^29^ The contributions of terms beyond linear order to the excitation energy, which are neglected in the linearized approximation, do not exceed 10^–3^ eV in the worst cases.

Concerning the use of a common ρ_B_ for all states, this approximation may affect the accuracy of excitation energies in the standard FDET protocol, but the extent of this effect depends on the specific choice of ρ_B_. If the chosen ρ_B_ satisfies the non-negativity condition for the total target density, it does not introduce additional error into the total energy as defined by the FDET energy expression.^4^ The relationship between violations of the non-negativity condition and resulting errors in the FDET total energy has been studied extensively^17^ showing that prepolarization often reduces the extent of the violation. However, verifying this condition requires knowledge of the exact total electron density for each electronic state—information that is typically unavailable in multiscale simulations. This makes such verification impractical.

There are no universal guidelines for selecting a common ρ_B_ when evaluating excited states. Our approach is to assess the sensitivity of excitation energies to different choices of ρ_B_, starting from the standard FDET protocol—i.e., the superposition of electron densities of individual solvent molecules.^21,22,30^ Such a sensitivity analysis can be performed on a case-by-case basis using small representative clusters of the solvated chromophore prior to a large-scale multiscale simulations.

In this context, it is important to address the interpretation of using a common ρ_B_ as implying that the effect on the excitation energies referred to in the literature as “state-specific solvent polarization” ^18,31,32^ is neglected. Within the framework of FDET, the concepts of “solvent polarization” and “state-specific solvent polarization” are not uniquely defined. This is because the same total electron density can be represented by different pairs of ρ_B_ and Ψ_A_, where Ψ_A_ is a solution of the FDET eigenvalue equation (eq 2).^8^ Therefore, solvent polarization arising from interactions with the chromophore is inherently ambiguous.

In certain approximated versions of FDET—such as those employing a simplified embedding potential ${ṽ}_{xct}^{nad}$ and the monomer expansion approximation (MEA)—the pair (ρ_B_, Ψ_A_) becomes unique, allowing one to define “solvent polarization” as the difference between the electron density of isolated solvent molecules and the ρ_B_ used in the model. A particularly meaningful choice in this context is ρ_B_^FnT^, obtained by simultaneous optimization of ρ_B_ and Ψ_A_ using the freeze-and-thaw procedure.^19^ The corresponding FDET working equations for this choice were first provided by Khait and Hoffmann,^18^ and later applied by Neugebauer and collaborators to investigate the impact of state-specific polarization—defined in this, admittedly arbitrary, manner.

As noted in ref 22, state-independent FDET calculations typically perform well, with excitation energy errors below 0.1 eV in most cases, with a few exceptions. One such exception is, for instance, the π → π* excitation in a *p*-nitroaniline–water complex yielded an error of 0.24 eV in the state-independent approach, which was reduced to 0.002 and 0.09 eV using two types of state-specific ρ_B_. However, in other cases, state-specific ρ_B_ led to worse results. Our own analysis similarly revealed that the improvement from using different ρ_B_ for different excited states is generally small and does not justify the additional computational cost—especially considering that it also introduces nonorthogonality between embedded wave functions. In ref 22, an expression for the excitation energy based on state-specific ρ_B_ was derived for a formulation in which embedded wave functions remain orthogonal. This expression is consistent with the Hohenberg–Kohn functional for the total energy up to second-order terms in density changes induced by interaction and excitation.

## Computational Details

### Calculation of Excited States

The algebraic diagrammatic construction (ADC) method in the second-order, ADC(2),^33,34^ was used to obtain the reference excitation energies as well as their FDET counterparts. The differences of the eigenvalues λ_A_^*j*^ – λ_A_^0^ in eq 2, which are equal to the vertical excitation energies if *E*_xct_^nad^[ρ_A_,ρ_B_] is linearized (eq 5). The reference results for the entire clusters were obtained also from ADC(2) calculations, applying the frozen core approximation with 27 frozen core orbitals and 47 frozen virtual orbitals.

### Cluster Model Used for Calculations of the Reference Spectra

Several FDET-based protocols were benchmarked against the reference results obtained from ADC(2) calculations applied for SCN^–^ + 20 H_2_O clusters. The reference UV–vis absorption spectra were computed by averaging stick spectra for the clusters broadened with normalized Gaussians having a full width at half-maximum of 0.25 eV. The 32 structures of the cluster were taken from the ab initio molecular dynamics (AIMD) simulation reported in refs 23 and 24 were used. An example snapshot is shown in Figure 2. The resulting reference spectrum thus corresponds to the hypothetical limit for FDET in which the exact bifunctional *v*_xct_^nad^[ρ_A_,ρ_B_] is used and the chosen ρ_B_ is such that the inequality ∀**r** ρ_AB_^*j*^(**r**) ≥ ρ_B_(**r**) holds, for all considered excited states *j* for the same cluster. We note that a similar procedure was used to generate the spectra in refs 23 and 24 , although a different method was used to evaluate vertical excitation energies (EOM-CCSD) and the SCN^–^ + 20 H_2_O cluster was embedded in the electrostatic field generated by water molecules outside of this cluster.

**Figure 2:**
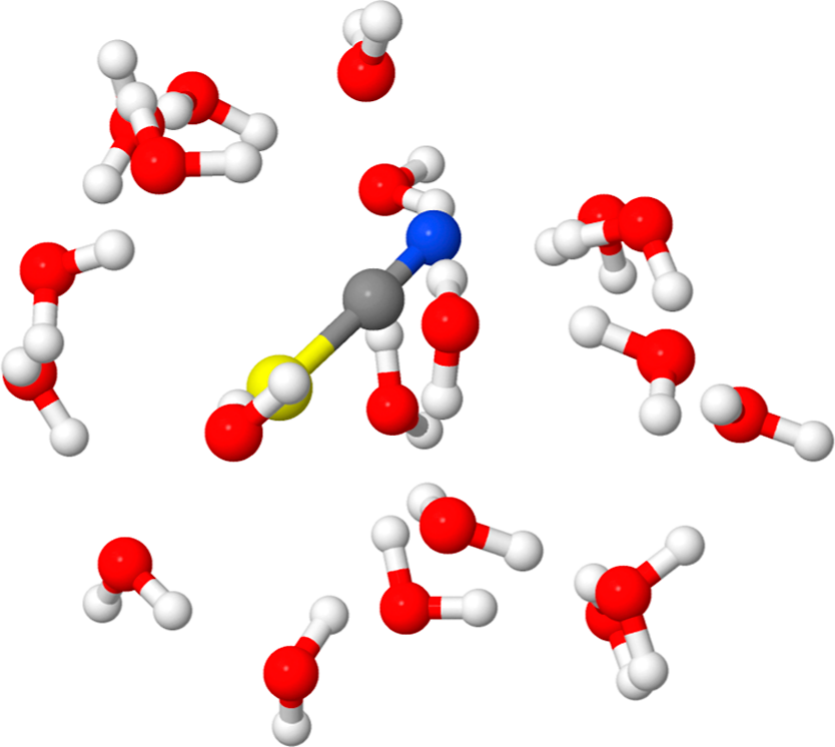
Geometry of SCN^–^ and 20 surrounding water molecules in one of the 32 snapshots used to model aqueous thiocyanate.

### Analysis of Excited States

In addition to oscillator strengths and excitation energies, we also use NTOs and exciton descriptors, such as the size of the hole and particle, or the average distance between the hole and particle,^35–40^ as interpretational tools to analyze the character of excited states. The term “exciton” refers to the transition density, which describes the difference between the initial and final electronic states involved in the transition in terms of one-electron transitions

6 $$\rho \left(r_{e},r_{h}\right)=\sum_{pq}\gamma_{pq}\phi_{p}\left(r_{e}\right)\phi_{q}\left(r_{h}\right)$$

where ϕ_*p*,*q*_ denote molecular orbitals and γ_*pq*_ is one-particle transition density matrix connecting the two states

7 $$\gamma_{pq}=\left\langle \Psi^{F}\left|p^{†}q\right|\Psi^{I}\right\rangle$$

Singular-value decomposition of γ yields NTOs, which provide most compact representation of the transition density. Transition density can be used to compute observables, such as oscillator strength, as well as the aforementioned exciton descriptors.

### FDET Calculations

In all FDET calculations, semilocal approximants for the functional *v*_xct_^nad^[ρ_A_,ρ_B_] defined in ref 4 were used. Conventionally, *v*_xct_^nad^[ρ_A_,ρ_B_] is split into exchange–correlation and kinetic parts. Each of them is due to nonadditivity of the density functionals known in the Kohn–Sham formulation^41^ of density functional theory (DFT):^27^ *E*_xc_[ρ] and *T*_s_[ρ], respectively. For the exchange–correlation component *v*_xc_^nad^[ρ_A_,ρ_B_], we used the decomposable local-density approximation: Slater–Dirac functional^42^ for exchange, and the Vosko–Wilk–Nusair parametrization^43^ of the correlation energy of uniform electron gas reported by Ceperley and Alder,^44^ whereas its *v*_c_[ρ_A_] part was entirely neglected in all FDET calculations. This approximant is denoted with *v*_xc_^nad(LDA)^[ρ_A_,ρ_B_]. For the *v*_*t*_^nad^[ρ_A_,ρ_B_] component of *v*_xct_^nad^[ρ_A_,ρ_B_] two approximants were considered: either *v*_*t*_^nad(LDA)^[ρ_A_,ρ_B_]­(**r**) a decomposable approximant derived from the Thomas-Fermi^45,46^ functional for the kinetic energy or a recently introduced nondecomposable semilocal approximant *v*_*t*_^nad(NDCS)^[ρ_A_,ρ_B_],^26^ which is expected to better describe embedding situations prone to artificial leakage of charge from the embedded species to the environment^47^ (such as cases where the electron detachment energy for the isolated embedded species is low).

Linearized FDET was applied for excited-states calculations.^7^ First-order Møller–Plesset corrected ground state Hartree–Fock density of the isolated chromophore, represented in a monomer expansion, was used as ρ_A_^ref^ in eq 5 in all cases. Unless otherwise stated, the environment density ρ_B_ was generated by the superposition of the density of each water molecule obtained by the Hartree–Fock method. The vertical excitation energies (ε), oscillator strengths (*f*), NTOs, and exciton descriptors were computed using the Q-Chem^48^ program in which FDET is implemented.

The use of a single reference nonvariational correlated method to evaluate differences of eigenvalues in eq 2 makes it possible to avoid using the functional for the correlation potential *v*_c_[ρ_A_] needed in case of variational methods (such as the methods considered in the original formulation of FDET^4^). Up to quadratic terms in the correlation induced change of density, the energy—which is equal to the one given by the Hohenberg–Kohn functional—can be obtained numerically without requiring either *v*_c_[ρ_A_] or the correlation energy functional *E*_c_[ρ_A_] (see the recently derived extension of FDET for nonvariational correlated methods, ref 5). The eigenvalues of eq 2 obtained from single reference nonvariational correlated methods are used instead. The use of the same method for correlation in FDET and the reference calculations allows us to attribute any deviation from the reference data solely to the approximations made in the used FDET-based method.

We used mixed basis sets, wherein the SCN^–^ and the 11 nearest water molecules (which we refer to as the first solvation shell) were described by the 6-31+G* basis sets and the remaining nine waters were described by the 6-31G basis sets.

Additional FDET calculations were performed using a hierarchy of increasingly supermolecular basis-set expansions for the embedded wave function. This hierarchy, referred to as SMEX, consists of successive augmentations of the basis set as follows:

- SME0: A monomer expansion augmented by the outer valence (most diffuse) *s* and *sp* shells from the 6-31+G* basis set on the hydrogen and oxygen atoms, respectively, of the first solvation shell surrounding the thiocyanate anion.
- SME1: SME0 supplemented by the second inner valence *sp* shells on the oxygen atoms and the inner *s* shells on the hydrogen atoms.
- SME2: SME1 further extended by adding the first inner valence *sp* shell on the oxygen atoms.
- SME3: SME2 expanded to include the core *s* basis functions on the oxygen atoms.

In the SMEX hierarchy, the *d* polarization shells on oxygen atoms were omitted to reduce computational costs. Their inclusion in the SME1 expansion results in the change in the FDET derived excitation energies of the order of only 0.1 meV, for instance.

These calculations were carried out using custom programs built with the PySCF^49^ and ADC-Connect^50^ Python packages to facilitate quick prototyping and flexible exploration of different FDET protocols; however, they can also be performed using the Q-Chem software package. An example input file and detailed instructions for performing analogous calculations using the Q-Chem software—excluding those involving the *v*_*t*_^nad(NDCS)^[ρ_A_,ρ_B_] approximant, which is not yet implemented in Q-Chem, are available at https://github.com/mingxueF/pyscf_qchem_Vemb.

For all calculations carried with the SMEX basis hierarchy, as well as for additional calculations using the MEA carried for comparison, the environment’s density ρ_B_ was generated by solving the Hartree–Fock equations for a system composed of 20 water molecules subjected to the electrostatic potential due to CHELPG net charges on S, C and N determined from the ground state density of an isolated thiocyanate at the given geometry. The density ρ_B_ was represented exclusively by basis functions centered on the water molecules’ atoms.

We note that, while the use of the SMEX expansion increases computational cost compared to the monomer expansion of the embedded wave function, it remains significantly more efficient than simply enlarging the embedded subsystem, because the number of electrons in the calculation does not increase. In ADC(2) calculations, the computational bottleneck lies in the transformation of electron–repulsion integrals from the atomic orbital (AO) basis to the molecular orbital (MO) basis. Although techniques such as Cholesky decomposition can reduce the formal scaling of this step, the canonical scaling is *N*_occ_ *N*_AO_^4^, where *N*_occ_ and *N*_AO_ are the numbers of occupied orbitals and atomic orbitals, respectively.

In the case of the embedded thiocyanate anion studied here, *N*_occ_ = 15 remains constant, while *N*_AO_ increases from 70 to 147 between the SME0 and SME3 expansions. This roughly corresponds to a 20-fold increase in computational cost. Nonetheless, this remains more affordable than embedding the entire first solvation shell, which would additionally raise *N*_occ_ from 15 to 70—resulting in an additional 5-fold increase in cost.

## Results and Discussion

We begin by analyzing the absorption spectrum of aqueous thiocyanate as described by the standard FDET protocol. This protocol employs a monomer expansion for the embedded thiocyanate, the representation of the environment density as a sum of isolated water fragment densities, and the *v*_*t*_^nad(LDA)^[ρ_A_,ρ_B_] bifunctional. It is applied to two choices of partitioning the SCN^–^ + 20 H_2_O cluster into one part represented by means of Ψ_A_ and another by ρ_B_. The first choice entailed including all 20 water molecules in ρ_B_, and the second choice entailed including only nine most distant water molecule in ρ_B_.

Building on the insights gained from our systematic benchmarks, we propose an improvement of the standard protocol to better capture the charge-transfer character of the excited states of aqueous thiocyanate. This involves examining the convergence of the absorption spectrum along a hierarchy of increasingly supermolecular expansions of the embedded system’s wave function, as well as assessing the effect of the two considered approximants for *v*_xct_^nad^[ρ_A_,ρ_B_].

To describe the character of the excited states, we used transition density matrix analysis.^35–40^ This analysis focuses on exciton size and includes visualizations of natural transition orbitals to gain insights into the nature of the electronic excitations.

### The Standard FDET Protocol

#### SCN^–^ Embedded in 20 Water Molecules

We first examine the absorption spectrum computed by the standard FDET protocol, which we treat as an entry-level method expected to fail in case of CTTS excitations for the reasons elaborated on in the Section Computational Details. This protocol uses the monomer expansion for the embedded wave function. It can be applied to the embedded system consisting of the chromophore alone or extended to include additional solvent molecules. In the first case, the standard protocol is expected to fail in describing CTTS excitations because the basis set does not contain functions centered on atoms in the solvent. We begin by analyzing this case.

Figure 3 shows the spectrum computed by the standard FDET protocol applied for two sizes of the part described by means of Ψ_A_. We do not show the spectrum of the isolated thiocyanate anion as this anion does not support bound excited states in the gas phase. The reference spectrum shows two major peaks at around 5.5 and 6.6 eV, respectively. The low energy peak can be attributed to two *s*-type CTTS excitation, whereas the high energy one is dominated by several excitations of mixed intramolecular and *p*-type CTTS characters; see Table 1 for NTOs of each excited state in a representative snapshot from AIMD simulations.

**Figure 3:**
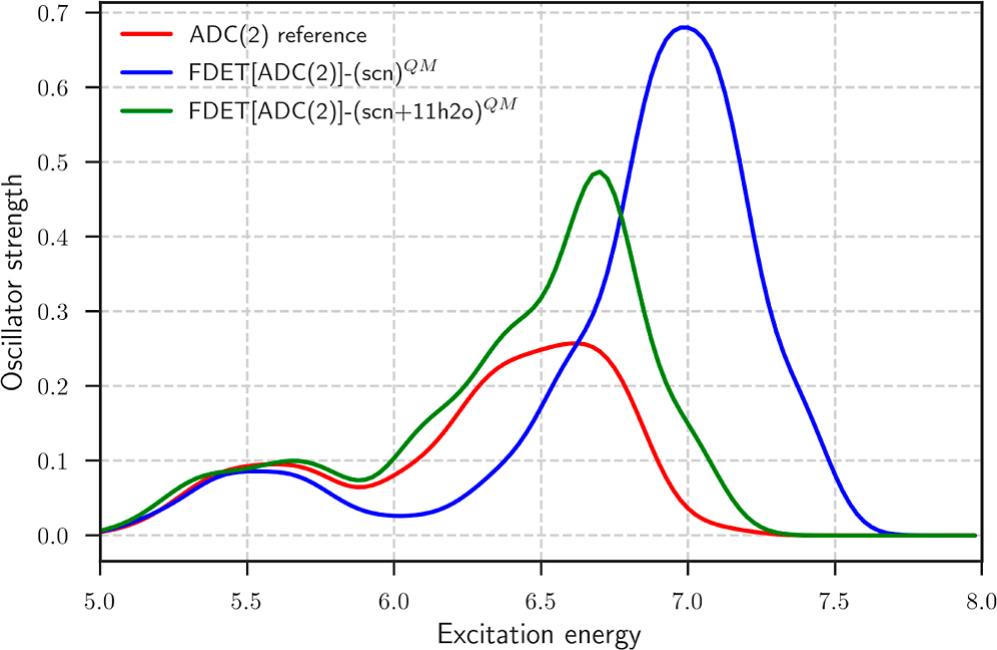
Absorption spectra computed by averaging over 32 AIMD snapshots. The red line represents the results from the ADC(2) reference calculations for the whole SCN^–^ + 20 H_2_O cluster, and the blue line represents the FDET results with embedded SCN^–^ and a frozen environment of 20 H_2_O molecules. The green line shows the FDET results with embedded SCN^–^ and its first solvation shell (11 H_2_O) along with a frozen environment of 9 H_2_O molecules. Energies are in eV. Results are obtained using the standard FDET protocol.

**Table 1:**
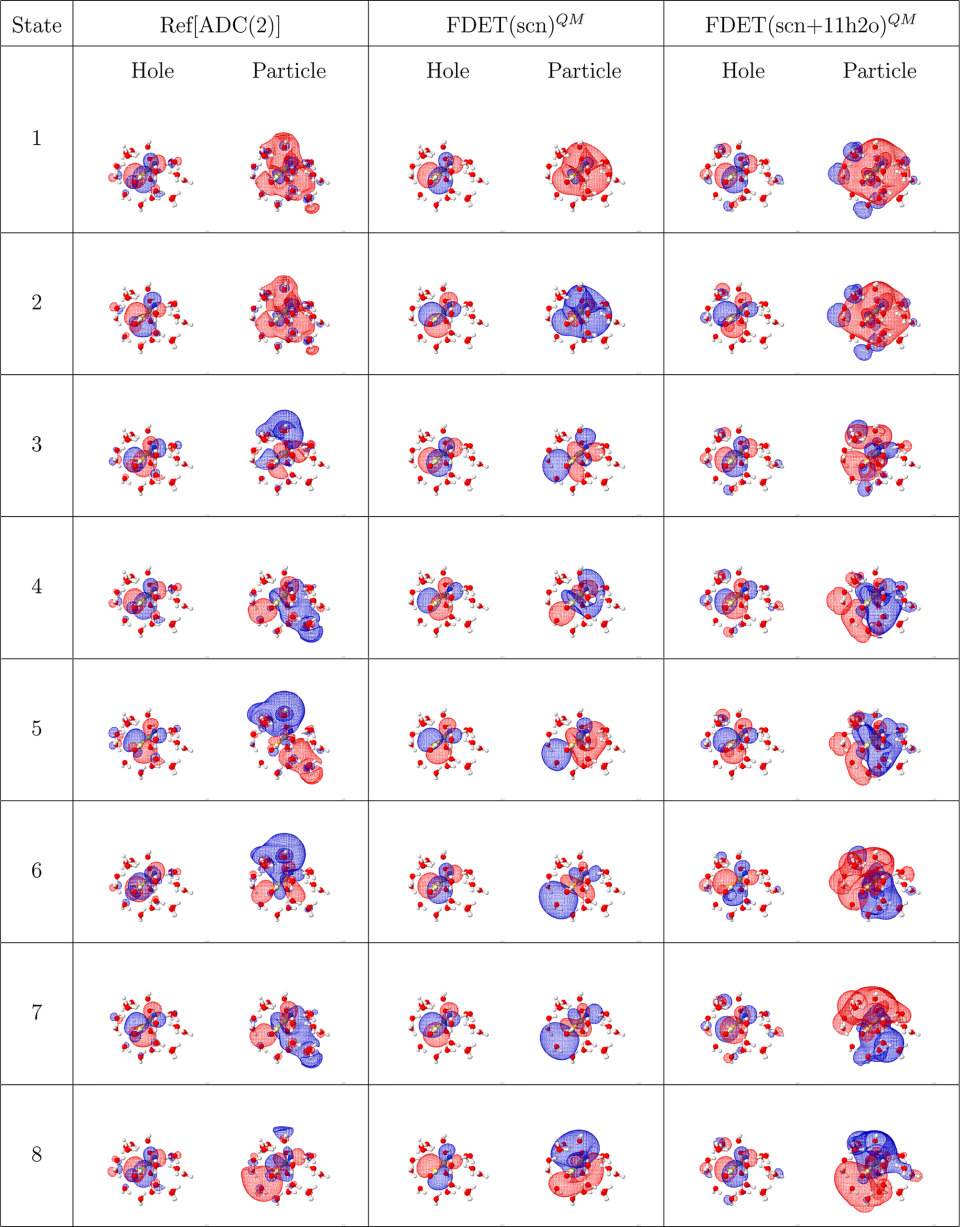
Dominant NTO Pairs (Hole and Particle Orbitals) for the Eight Lowest Excited States, Obtained from the Reference ADC(2) Calculations and the Standard FDET Protocols with Either Only SCN^–^ (Central Column) or SCN^–^ + 11 H_2_O (Right Column) Included in ${Ĥ}_{A}$ ^a^

a An isovalue of 0.015 a.u. was used to plot the NTO isosurfaces. For excitation energies and oscillator strengths, see Table S1 in the Supporting Information.

Figure 3 shows that the standard FDET protocol describes the low energy absorption peak surprisingly well, despite the delocalized character of the excited states even if only SCN^–^ is represented by an embedded wave function. To assess how well the FDET standard protocol captures the effects of Pauli repulsion on excitation energies, we compared its performance to two point charge schemes used to model the environment of 20 water molecules. The first one, based on Gaussian-smeared point charges (taken from the CHARMM27 force field), is designed to mimic electron delocalization in the real electron density. However, this approximation fails to accurately describe the lower-energy excitations, resulting in a significant red shift of approximately 0.4 eV (see Figure 4). In contrast to CHARMM27, using CHELPG-derived charges, which are fitted to the electrostatic potential of the environment, leads to a notable improvement, reducing the excitation energy error by 0.2 eV. The latter can be attributed to the error due to the neglect of the Pauli repulsion. It is clear that embedding makes a difference.

**Figure 4:**
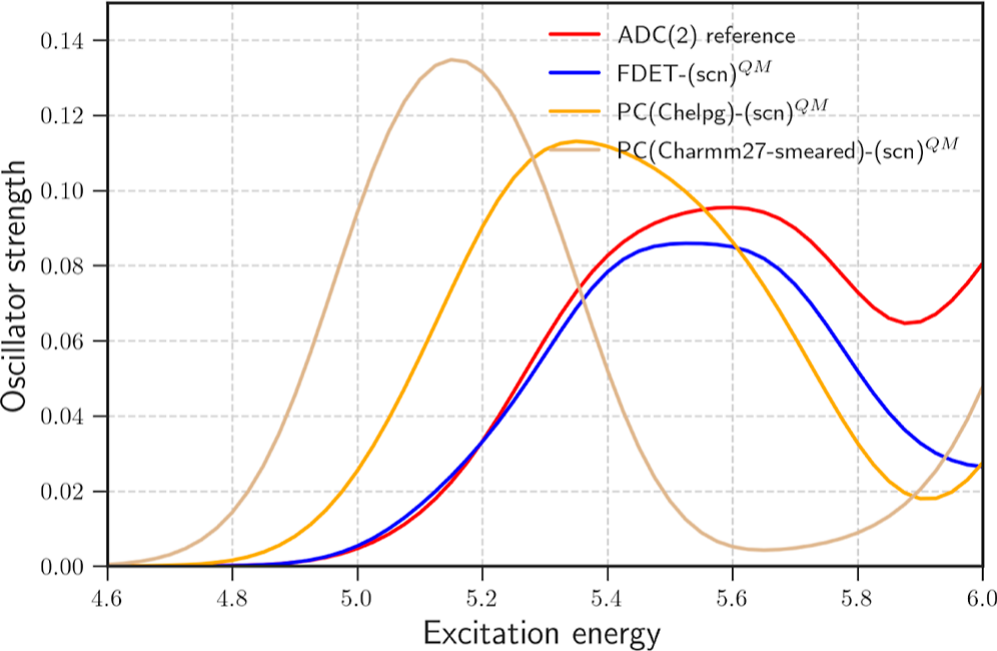
Spectra computed with different models of the environment (20 H_2_O molecules): the red line is the ADC(2) reference; the blue line shows FDET results with embedded SCN^–^ and a frozen ρ_B_; the orange line shows embedding with CHELPG point charges; and the gray line shows results with smeared CHARMM27 point charges. Energies are given in eV. The standard FDET protocol was used.

The two lower-energy excitations exhibit an *s*-like CTTS character. Gaussian-blurred point charges CHARMM27 inadequately attenuates the attractive nuclear potential, leading to an artificial stabilization of excited states and thus a systematic red shift in excitation energies. For this system, the CHELPG-derived charges provide a more accurate representation of the electrostatic environment, resulting in better agreement with reference calculations.

In contrast to both point–charge-based approaches, FDET embedding effectively accounts for Pauli repulsion via *v*_xct_^nad^[ρ_A_,ρ_B_] (see Figure 4). This prevents the artificial delocalization of electron density into the nuclear potential of the environment, leading to excitation energies that closely match the reference values. These results highlight the limitations of point charge approximations in capturing key effects governing excited-state properties and underscore the advantages of an FDET-based description.

As shown in Figure 3, the discrepancies between the FDET and reference spectra become increasingly pronounced at higher excitation energies. In particular, the higher-energy peaks in the FDET spectrum exhibit a blue shift of approximately 0.4 eV, accompanied by oscillator strengths that are nearly three times larger than those obtained from the reference ADC(2) calculations for the whole cluster. This substantial overestimation of intensities and shifts in peak positions suggests a fundamental limitation in the standard protocol’s treatment of high-lying excitations if only SCN^–^ is represented by the embedded wave function and ρ_B_ represents all the 20 water molecules.

To gain further insight into these deviations, we analyze the NTO for the corresponding excitations. Table 1 compares NTOs for the eight lowest excited states obtained from the reference ADC(2) and from FDET calculations for a representative molecular snapshot. A clear discrepancy emerges in the character of the reference particle NTOs for the highest excitations, which exhibits a *p*-like shape delocalized over the first solvation shell. In contrast to lower excitations, the FDET NTOs fail to capture this delocalization and instead display a predominantly intramolecular character, with limited delocalization to the water, as illustrated for a selected excitation in a single snapshot in Figure 5. This discrepancy arises from the monomer expansion of the wave function, which inherently restricts electronic transitions to the embedded subsystem and prevents the necessary delocalization onto surrounding water molecules.

**Figure 5:**
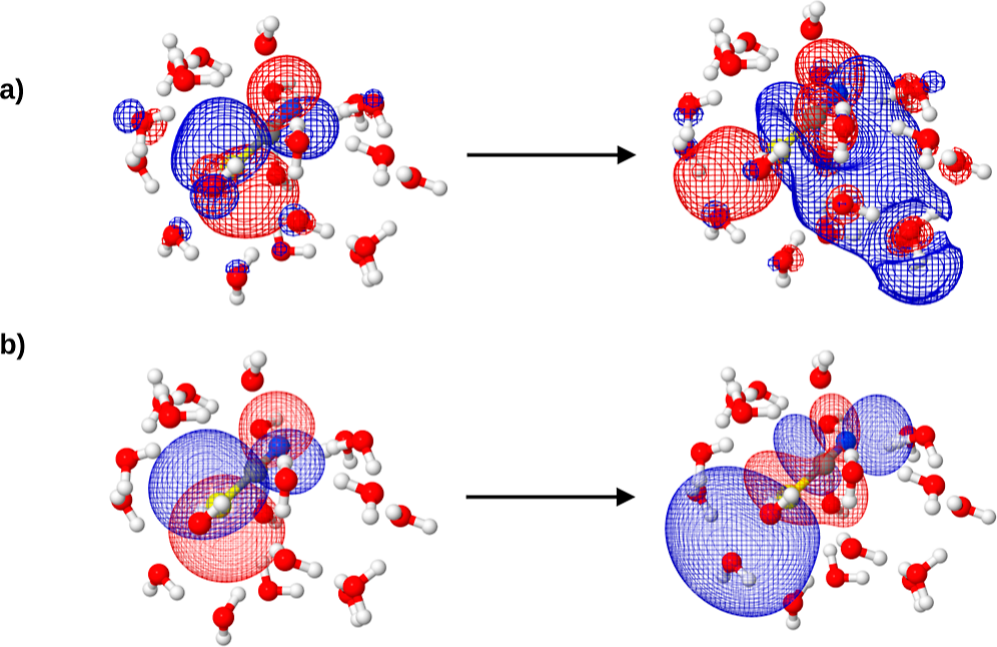
NTOs for a typical higher (seventh) excited state in an example snapshot: (a) reference ADC(2) calculation for the whole cluster and (b) FDET with the standard protocol.

Figure 6 provides a complementary perspective on these errors via the analysis of exciton sizes, where the mean values and standard deviations of the exciton size for the first 8 excited states are calculated across all snapshots.

**Figure 6:**
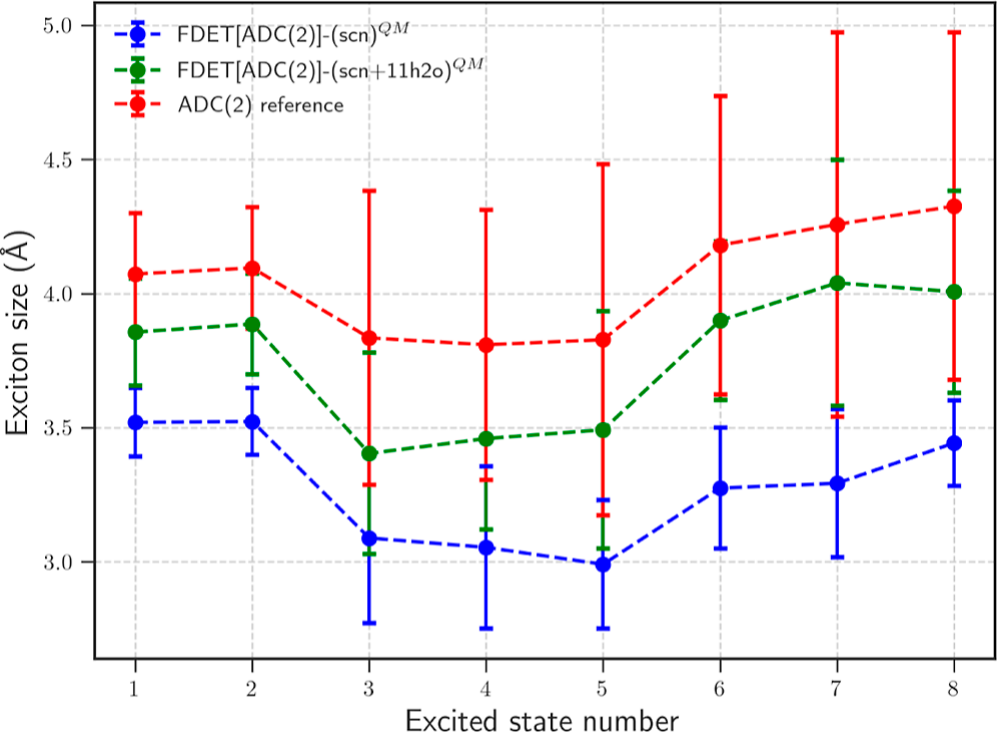
Mean values and standard deviations of the exciton size (in Å) for the 8 lowest excited states obtained from the set of 32 snapshots. See also the caption to Figure 3.

Examining the exciton size trends across different states, the excited states can be categorized into three groups. The first group, comprising the two lowest excited states, exhibits a moderately extended exciton size, characteristic of an *s*-type CTTS transition. The second group, which includes the next three states, is characterized by smaller exciton sizes, indicative of intramolecular transitions localized within SCN^–^. The third group, consisting of the next three states, exhibits the largest exciton sizes, corresponding to *p*-type CTTS transitions where the electronic density is more extensively delocalized over the solvent environment. The FDET calculations with only SCN^–^ as the embedded subsystem reproduce the overall trend of exciton sizes observed in the reference, though with significant deviations for certain states. We observe a strong correlation between the accuracy of exciton size predictions and the overall accuracy of the computed spectra. States with significant errors in exciton size are also those with the largest deviations in excitation energies and oscillator strengths. These findings highlight the importance of properly accounting for the electronic delocalization of higher excitations, which is not adequately captured by the standard FDET protocol with MEA. In the following sections, we explore two possible ways to overcome the failure of the standard FDET protocol. The first is a brute-force one and consists of increasing the size of the system described by means of the embedded wave function (adding solvent molecules to the quantum subsystem). The other one limits the use of the embedded wave function to SCN^–^ but goes beyond the approximations used in the standard FDET protocol.

#### SCN^–^ + 11 H_2_O Embedded in 9 Water Molecules

The analysis of the failures of the standard protocol suggests an obvious approach to improve the FDET treatment of the absorption spectrum is to apply the standard protocol to an *effective chromophore*, consisting of the thiocyanate anion and its neighboring water molecules.

As seen in Figure 3, this extended embedded region provides excitation energies much closer to the ones from the reference ADC(2) calculations for the entire cluster. In particular, the peak position related to the higher excited states obtained with FDET­(SCN^–^ + 11 H_2_O)^QM^ show good agreement with the reference calculations. The effect of embedding the full first solvation shell is not only limited to improved peak positions; it also plays a crucial role in mitigating spuriously high-intensity states that were present when only the SCN^–^ anion was embedded. The presence of these spurious states in smaller embedded regions suggests that the electronic coupling between SCN^–^ and the surrounding water molecules is not fully captured when only the chromophore is embedded. By explicitly including the full solvation shell, these interactions are described better, leading to a more physically meaningful absorption spectrum. These improvements in the spectrum are closely linked to changes observed in the NTOs. As shown in Table 1, the delocalization of excited states over the solvent is restored across all excitations, and the particle NTOs of the higher excited states recover their characteristic *p*-like shape. Furthermore, as illustrated in Figure 6, the systematic underestimation of exciton sizes observed with the standard protocol using monomer expansion is largely corrected when the embedded subsystem is extended, bringing the results into much better agreement with the reference.

Despite these improvements, the oscillator strength for the higher peak remains significantly overestimated in the FDET­(SCN^–^ + 11 H_2_O)^QM^ scheme. This might be attributed to the fact that the embedded system, even when extended to include the first solvation shell, does not capture all relevant orbitals contributing to the electronic transitions. Some excited states may involve orbitals localized on water molecules beyond the first solvation shell, which remain frozen in FDET calculations. These missing contributions can lead to discrepancies in oscillator strengths and transition characters, particularly for higher excited states. Although the extended embedded region mitigates this issue to some extent, the exclusion of additional solvent orbitals remains a limitation of the approach.

The brute-force attempt to extend the range of applicability of the standard FDET protocol by increasing *N*_A_ by 110 and decreasing *N*_B_ by the same number of electrons not only involves significant computational cost but does not lead to satisfactory agreement between the FDET and reference spectra.

### Beyond the Standard Protocol: Refinements of the Embedding Treatment

Rather than expanding the embedded subsystem, we now explore how much improvement can be achieved while keeping only SCN^–^ as the embedded system and refining the standard protocol. Three key modifications are considered: first, we assess whether prepolarizing the environment density, ρ_B_, by the electrostatic field of the embedded species in its ground state enhances the accuracy of excitation energies. Second, we investigate the effect of supermolecular expansion of the embedded wave function, wherein the basis set of the environment is included in the wave function expansion of the embedded system. Since the success of this approach depends on the accuracy of the approximant to *v*_*t*_^nad^[ρ_A_,ρ_B_] at the interface, we simultaneously address the limitations of the used approximants by comparing the results obtained by means of the standard one *v*_*t*_^nad(LDA)^[ρ_A_,ρ_B_] to results obtained using a more apt bifunctional, *v*_*t*_^nad(NDCS)^[ρ_A_,ρ_B_].

This section presents a detailed analysis of these refinements, examining the extent to which they improve the accuracy of excitation energies while maintaining a computationally feasible embedded subsystem.

### Prepolarization of the Environment

A common first step toward improving the standard FDET protocol in cases where electronic interactions between the chromophore and the solvent are significant is to prepolarize the density of the environment. This is achieved by optimizing ρ_B_ in the presence of the embedded chromophore’s electrostatic potential. In principle, as long as ρ_B_ satisfies the non-negativity condition ρ_AB_ ≥ ρ_B_, the exact FDET energies should be independent of the choice of ρ_B_. The polarization of the environment in the presence of the embedded system should therefore be accounted for implicitly. However, the approximate functionals used in practice for *v*_xct_^nad^[ρ_A_,ρ_B_] do not guarantee an accurate implicit treatment of environmental polarization. Moreover, determining in advance whether a given choice of ρ_B_ violates the non-negativity condition is not straightforward.^17^ Here, we apply the prepolarization strategy to our system and evaluate its impact on the predicted absorption spectrum.

Figure 7 presents the absorption spectrum obtained from the standard protocol with ρ_B_ constructed as the sum of the densities of 20 isolated water molecules is compared to that obtained when ρ_B_ is prepolarized by subjecting the 20 water molecules to the electrostatic potential generated by the CHELPG charges of the ground-state density of SCN^–^. Prepolarization appears to stabilize the ground state relative to the excited states, leading to an additional blue shift of approximately 0.1 eV in the absorption spectrum. This is expected, since the polarization of the environment by the ground state, in which the charge is more localized, should be more important than for the excited states, where the charge is more diffused. Also, the violation of the non-negativity condition is expected to be more important for the ground state. Moreover, the oscillator strength of the highest-energy peak is further overestimated, exacerbating the discrepancy with the reference calculations.

**Figure 7:**
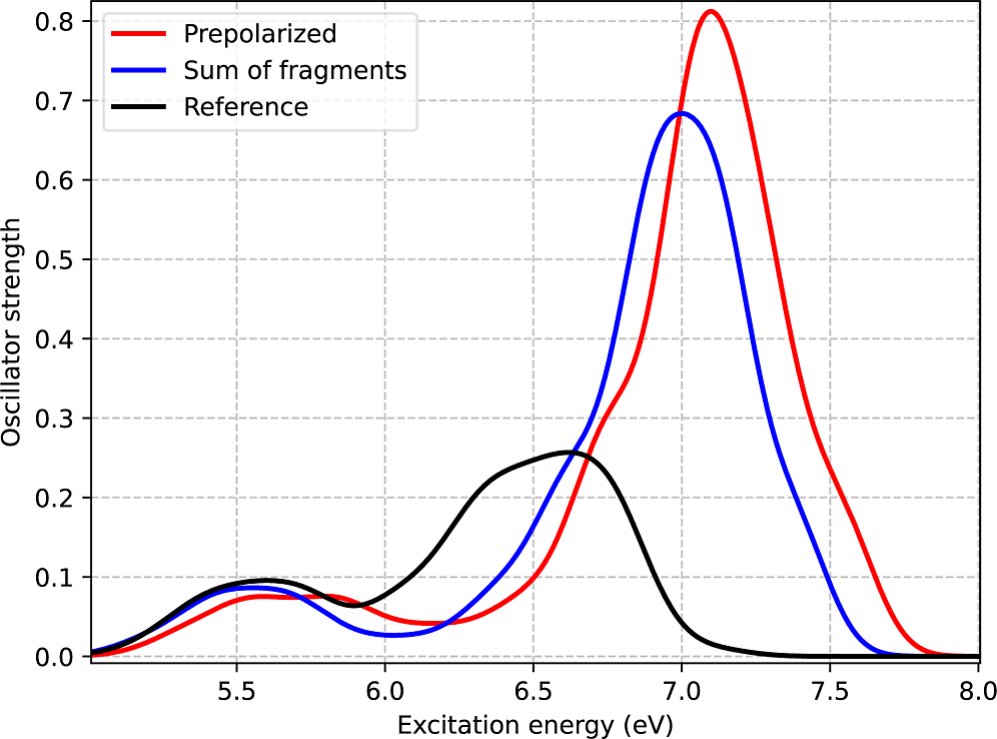
Absorption spectra computed by averaging over 32 AIMD snapshots obtained using different methods for vertical excitation energies in the SCN^–^ + 20 H_2_O cluster: ADC(2) reference (shown in black) together two FDET protocols for embedded SCN^–^ either with (red) or without (blue) polarization of ρ_B_. For FDET calculations, the monomer expansion was used, along with the *v*_xct_^nad(LDA)^[ρ_A_,ρ_B_] approximant.

These results suggest that the seemingly good agreement obtained by the standard protocol for the lowest-energy peak may, in part, be due to error cancellation. Specifically, the lack of stabilization of the ground state that would be introduced by prepolarization could counterbalance the lack of stabilization of the excited states resulting from limitations of the monomer expansion.

Ultimately, prepolarization alone is insufficient to improve the description of the spectrum in this system. However, since the subsequent refinements primarily aim to improve the accuracy of excited states’ energies rather than the ground state energy we cannot rely on the same error cancellation mechanism seen in the standard protocol (with ρ_A_ representing the density of SCN^–^) between ground and excited states. Therefore, prepolarization is retained in all further calculations that will assess the impact of further refinements such as supermolecular expansion and the choice of the approximant for *v*_*t*_^nad^[ρ_A_,ρ_B_].

### Beyond Monomer Expansion Approximation in FDET

As discussed in the previous subsections, the poor description of CTTS excitations within the standard FDET protocol stems from the use of monomer-based basis set expansions, which inherently prevent charge transfer to the environment. Whereas a supermolecular expansion would allow such transfer, it also introduces the risk of unphysical charge delocalization into the environment, depending on the quality of the approximant used for *v*_*t*_^nad^[ρ_A_,ρ_B_].

To investigate this issue, we employ a hierarchy of increasingly supermolecular basis set expansions, denoted as SMEX and described in the Section Computational Details, in conjunction with two different approximants for *v*_*t*_^nad^[ρ_A_,ρ_B_]: *v*_*t*_^nad(LDA)^[ρ_A_,ρ_B_] and *v*_*t*_^nad(NDCS)^[ρ_A_,ρ_B_].^26^

We now turn to the analysis of the convergence behavior of excitation energies as the supermolecular expansion is systematically extended for both approximants. Figures 8 and 9 illustrate the evolution of the mean excitation energies over 32 AIMD snapshots for the 8 lowest excited states of aqueous thiocyanate along the SMEX hierarchy of basis sets, where the degree of supermolecular character is progressively increased.

**Figure 8:**
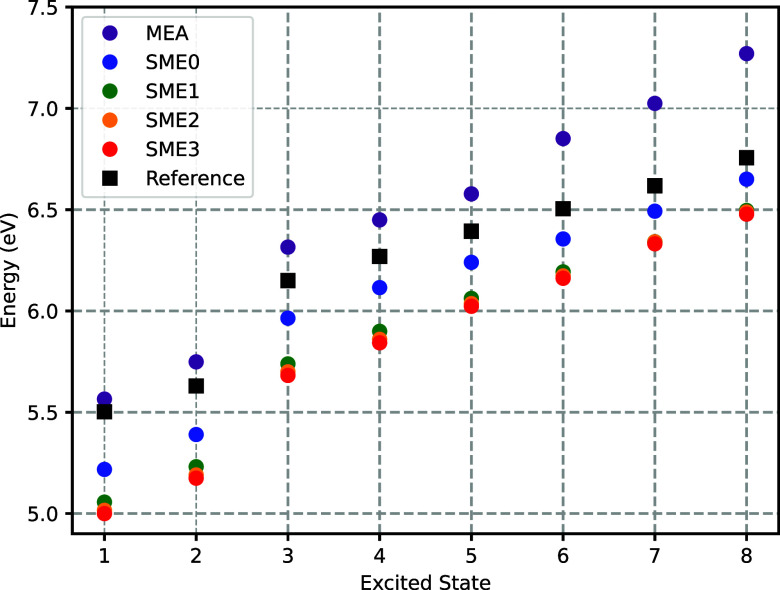
Convergence of the 8 lowest FDET vertical excitation energies calculated for embedded SCN^–^ using the *v*_xct_^nad(LDA)^[ρ_A_,ρ_B_] approximant along the SMEX supermolecular expansion hierarchy. The reference ADC(2) results for the SCN^–^ + 20 H_2_O cluster are shown in black. The dots and squares show the mean over 32 AIMD snapshots.

**Figure 9:**
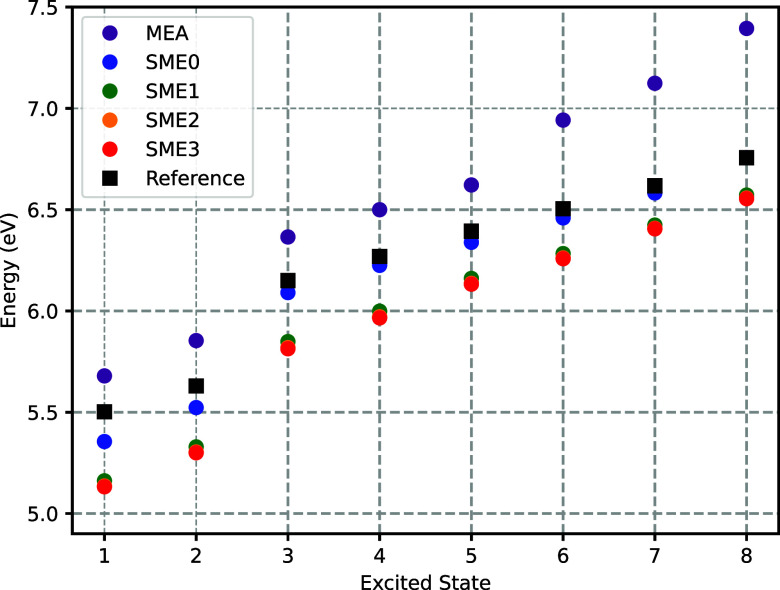
Mean excitation energies as in Figure 8 but obtained with the *v*_*t*_^nad(NDCS)^[ρ_A_,ρ_B_] approximant instead of *v*_*t*_^nad(LDA)^[ρ_A_,ρ_B_].

Upon the addition of the most diffuse (outer) valence shell centered on the atoms of the water molecules, both approximations yield excitation energy predictions that are significantly lower than the results obtained with a monomer expansion. For *v*_*t*_^nad(NDCS)^[ρ_A_,ρ_B_], this amounts to excitation energies that are significantly and systematically closer to the reference ADC(2) results, as shown in Figure 10. For *v*_*t*_^nad(LDA)^[ρ_A_,ρ_B_], however, the effect varies from a deterioration of the error by about 0.3 eV for the lowest excited state to the improvement of about 0.4 eV for the highest excited state.

**Figure 10:**
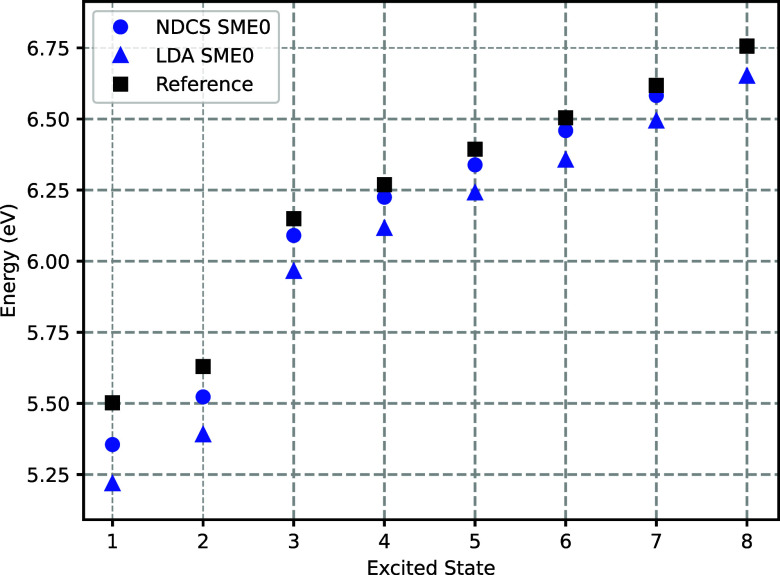
Comparison of the mean excitation energies obtained with *v*_*t*_^nad(NDCS)^[ρ_A_,ρ_B_] and with *v*_*t*_^nad(LDA)^[ρ_A_,ρ_B_]. Details of the SME0 basis set expansion are given in the Section Computational Details. See also captions to Figures 8 and 9.

As the basis set is further augmented along the SMEX hierarchy, and more compact (inner) valence shells as well as the core–shell are included, both approximations exhibit a substantial drop of approximately 0.25 to 0.4 eV in excitation energies. This trend ultimately leads to a systematic underestimation of excitation energies relative to the reference values. Given that *v*_*t*_^nad(NDCS)^[ρ_A_,ρ_B_] provides excitation energy predictions that are more realistic at convergence, we favor this approximant in the remainder of the discussion.

As shown in Figure 11, employing a partial supermolecular expansion (SME0) of the embedded wave function in conjunction with the *v*_*t*_^nad(NDCS)^[ρ_A_,ρ_B_] bifunctional significantly improves the computed electronic spectrum of the thiocyanate anion (SCN^–^) in aqueous solution compared to the MEA. This improvement is particularly evident in the excitation energies and oscillator strengths of the second peak, which corresponds to transitions of intramolecular and *p*-type CTTS character.

**Figure 11:**
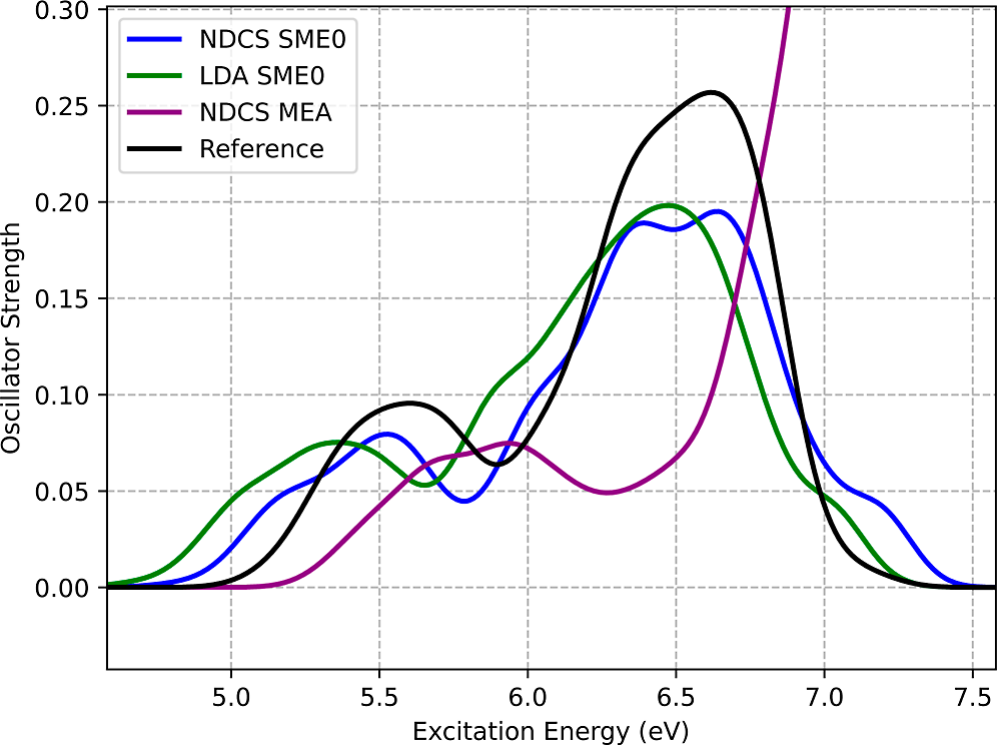
Absorption spectra computed by averaging over 32 AIMD snapshots obtained using different methods for vertical excitation energies in the SCN^–^ + 20 H_2_O cluster. ADC(2)—the reference in black, and three FDET protocols for embedded SCN^–^: (a) monomer expansion approximation using *v*_*t*_^nad(NDCS)^[ρ_A_,ρ_B_] (purple), (b) SME0 expansion of the basis set and *v*_*t*_^nad(NDCS)^[ρ_A_,ρ_B_] (blue), (c) SME0 expansion of the basis set and *v*_*t*_^nad(LDA)^[ρ_A_,ρ_B_] (green). Local density approximation was used for the *v*_xc_^nad(LDA)^[ρ_A_,ρ_B_] component of *v*_xct_^nad(LDA)^[ρ_A_,ρ_B_] in all FDET calculations.

For the first absorption peak’s position, the SME0 expansion leads to an underestimation of approximately 0.13 eV, whereas the monomer expansion, along with prepolarization and the *v*_*t*_^nad(NDCS)^[ρ_A_,ρ_B_] bifunctional, results in an overestimation of about 0.25 eV. However, for the second peak, the errors are effectively reduced: the overestimation of approximately 0.6 eV with MEA is replaced by an underestimation of about 0.1 eV with SME0. The oscillator strengths are also significantly improved, decreasing from intensities exceeding 300% of the reference value with MEA to approximately 70% with SME0.

The improvements observed in the spectrum are in line with the improvements in the exciton sizes shown in Figure 12.

**Figure 12:**
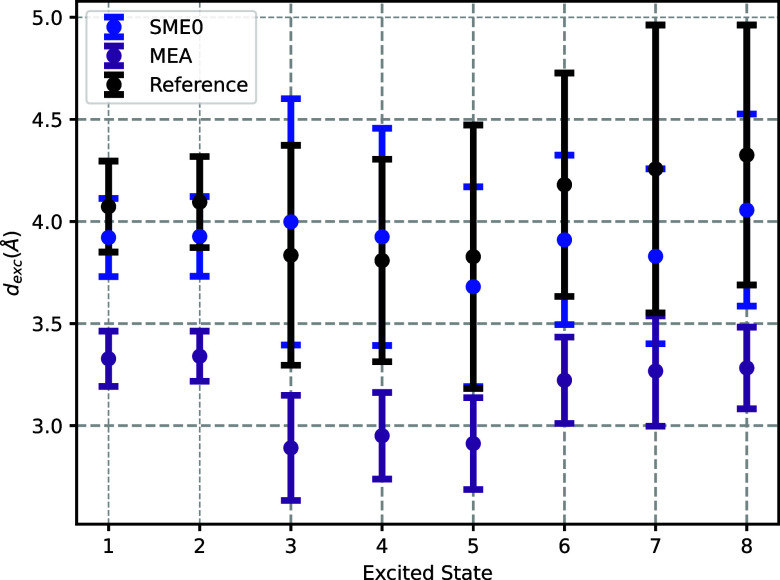
Mean and standard deviations over 32 AIMD snapshots of the exciton sizes for the 8 lowest excited states obtained using different methods for vertical excitation energies in the SCN^–^ + 20 H_2_O cluster. ADC(2)—the reference in black, and two FDET protocols for embedded SCN^–^: (a) monomer expansion approximation (purple), and (b) SME0 expansion of the basis set (blue). Local density approximation was used for the *v*_xc_^nad^[ρ_A_,ρ_B_] component of *v*_xct_^nad^[ρ_A_,ρ_B_] and NDCS for the *v*_*t*_^nad^[ρ_A_,ρ_B_] component in all FDET calculations.

These observations rise the question about the apparently better predictions obtained using the supermolecular expansion with the *v*_*t*_^nad(NDCS)^[ρ_A_,ρ_B_] bifunctional and 20 frozen water molecules, compared to the standard protocol with only 9 frozen water molecules. The analysis of NTOs provides valuable insight. In the reference calculations for the whole cluster, excitations involve some degree of electron removal from the first solvation shell (nearest 11 water molecules; see Table 1). In the scheme where all waters are frozen, this is not possible, whereas it is possible in the standard protocol with only 9 frozen waters. As a result, in calculations where all waters are frozen, the hole NTO sizes are likely underestimated. At the same time, in the standard protocol where the embedded system contains 11 water molecules, the particle NTOs only extend as much as those in calculations employing partial supermolecular expansions with all waters frozen. Consequently, the overlap between hole and particle NTOs—and therefore the oscillator strengths—is likely overestimated in FDET­(SCN^–^ + 11 H_2_O)^QM^ calculations and underestimated in FDET­(SCN^–^)^QM^ calculations with supermolecular expansions. This interpretation aligns with the observed trends in exciton sizes for both methods, which tend to be underestimated in FDET­(SCN^–^ + 11 H_2_O)^QM^ calculations and greater in FDET­(SCN^–^)^QM^ calculations with SME.

Despite the improvements gained by the augmented protocol, some discrepancies remain. The overestimation of exciton sizes for the third and fourth excited states—where the reference indicates an intramolecular excitation character—as well as the small fraction of intramolecular character in the NTOs, suggest a slight tendency to overdelocalize the electrons in the solvent. This behavior indicates potential deficiencies in the *v*_*t*_^nad(NDCS)^[ρ_A_,ρ_B_] bifunctional.

## Summary and Conclusions

We have investigated the performance of strategies within the FDET framework for modeling CTTS excitations in aqueous thiocyanate (SCN_aq_^–^). This type of problem typically requires the inclusion of solvent molecules in the embedded quantum subsystem to achieve accurate results. In this work, we explored how FDET-based methods can be adapted to tackle such problems without enlarging the size of the subsystem described at the wave function level. In our case limiting it to just the SCN^–^ anion.

In the first part, we confirmed the rather expected result that the conventional semilocal approximations for the nonadditive exchange-correlation-kinetic potential *v*_xct_^nad^[ρ_A_,ρ_B_], which have been successfully applied to local excitations, fail in the case of CTTS transitions due to significant electron density overlap between the chromophore and the solvent environment. This failure concerns in particular the standard FDET protocol, which employs a monomer expansion for the embedded system, a frozen environment density constructed from isolated fragment densities and to *v*_*t*_^nad(LDA)^[ρ_A_,ρ_B_] bifunctional. Our results indicate that while this protocol provides a reasonable description of low-energy excitations, it significantly overestimates the excitation energies and oscillator strengths of higher-energy transitions due to an inadequate treatment of electronic delocalization.

To improve the description of CTTS excitations within FDET, we explored the effect of extending the embedded region to include the first solvation shell of SCN^–^, which improved agreement with reference ADC(2) calculations for the entire cluster, particularly for higher-energy excitations. Additionally, we tried refinements of the standard protocol, including:

- Prepolarizing the environment density ρ_B_ using the electrostatic potential of the embedded chromophore, which did not by itself improve predictions for the absorption spectrum.
- Implementing a supermolecular expansion (SMEX) of the embedded wave function, allowing for greater electronic delocalization into the solvent. This approach to represent ρ_A_, combined with the improved approximant (*v*_*t*_^nad(NDCS)^[ρ_A_,ρ_B_] instead of *v*_*t*_^nad(LDA)^[ρ_A_,ρ_B_]), significantly enhanced the accuracy of excitation energies and oscillator strengths.

Our results underscore the interplay between the basis set used to describe the embedded system, the choice of ρ_B_, and the approximant for *v*_xct_^nad^[ρ_A_,ρ_B_] determining the accuracy of FDET-based predictions. We emphasize that improving one of these factors in isolation does not necessarily lead to systematic improvements, as their effects are interdependent. Therefore, practitioners should carefully consider how modifications to one approximation used in a give FDET protocol may influence other parameters of the model. The advantages of the *v*_*t*_^nad(NDCS)^[ρ_A_,ρ_B_] approximant over *v*_*t*_^nad(LDA)^[ρ_A_,ρ_B_] show most clearly if the basis set includes the atomic functions localized on the water molecules.

This work also highlights a central dilemma in FDET-based modeling of CTTS excitations: the need to extend the basis set into the solvent region to describe charge-transfer states accurately, weighed against the increased sensitivity to errors in the nonadditive kinetic energy potential *v*_xct_^nad^[ρ_A_,ρ_B_] that such extensions introduce. When the basis set is restricted, CTTS states cannot be properly described. When it is extended, however, the inaccuracies of current approximants—particularly in regions of density overlap—can compromise results. Our aim was to evaluate how well existing approximations perform under these competing demands. We hope that our work can serve as a benchmark for current FDET-based methods and help define where further improvements in functionals are most needed.

We stress that the extensions to the standard FDET protocol presented here should not be seen as a general replacement or invalidation of the standard FDET protocol. Rather, they constitute a strategy for addressing the specific challenge of modeling CTTS excitations, which clearly lie outside the regime where the standard protocol has proven effective. The refinements proposed here—like our earlier work using state-specific ρ_B_(**r**) densities^22^—should be understood as complementary and problem-specific adaptations, offering a flavor of how the protocol can be adapted in challenging cases.

Beyond the immediate implications for FDET-based calculations of CTTS excitations, this study highlights persistent challenges associated with the approximants for *v*_xct_^nad^[ρ_A_,ρ_B_]. The observed discrepancies serve as a valuable diagnostic for identifying limitations in existing approximants and guiding the development of more accurate functionals. Until a more reliable and universally accurate approximant for *v*_xct_[ρ_A_,ρ_B_] is available, caution is warranted in expanding the flexibility of the embedded wave function, particularly by adding functions localized in the environment. Although an initial inclusion of such functions improves the quality of excitation energies and oscillator strengths, further additions may lead to overdelocalization and diminished accuracy. Notably, even when the supermolecular expansion exceeds the optimal range, FDET remains numerically stable and the deterioration in accuracy is moderate. This robustness reinforces the value of FDET as a reliable embedding framework despite current limitations in approximate functionals.

## Supplementary Material



## Notes

Transition energies, oscillator strengths and the geometry for the snapshot shown in Figure 2, averages and standard deviations of transition energies and oscillator strengths over all the used snapshots (PDF)

## ⊥

Department of Energy Conversion and Storage, Technical University of Denmark, Agnes Nielsens Vej, 301, 2800 Kgs. Lyngby, Denmark
