# Supplementary material for: Can Semilocal Approximations to the Embedding Potential Tackle Charge-Transfer-to-Solvent Excitations? An Aqueous Thiocyanate Example
Source: J Chem Theory Comput. 2025 Oct 6;21(20):10452–65. doi: 10.1021/acs.jctc.5c00991 (PMC12573756; doi:10.1021/acs.jctc.5c00991)
Supplement: Supplementary file 1 [file ct5c00991_si_001.pdf]

Supporting information for: Can semi-local approximations to the embedding potential tackle charge-transfer-to-solvent excitations? An aqueous thiocyanate example.

Pierre-Olivier Roy,[†] Mingxue Fu,[‡] Ronit Sarangi,[¶,§] Anna I. Krylov,[*,§] and Tomasz A. Wesolowski[*,†]

†*Université de Genève, Départment de Chimie Physique 30, Quai Ernest-Ansermet, CH-1211 Genève 4, Switzerland*

‡ *Department of Energy Conversion and Storage, Technical University of Denmark, Agnes Nielsens Vej, 301, 2800 Kgs. Lyngby, Denmark*

¶*Department of Chemistry, Syracuse University, Syracuse, NY 13244, USA*

§*Department of Chemistry, University of Southern California, Los Angeles, California 90089-0482, USA*

E-mail: krylov@usc.edu; tomasz.wesolowski@unige.ch

Table S1: Excitation energies (in eV) and oscillator strengths f results for the eight lowest excited states, obtained from the reference ADC(2) calculations and the standard FDET protocols with either only SCN^- or $SCN^- + 11H_2O$ included in $\hat{H}_A$. The natural transition orbitals of corresponding excited states are shown in Table 1 in the main text.

State	$FDET(SCN^-)^{QM}$		$FDET(SCN^-+11H_2O)^{QM}$		Ref[ADC(2)]	
	ϵ	f	ϵ	f	ϵ	f
1	5.514	0.0154	5.438	0.0164	5.429	0.0126
2	5.611	0.0135	5.552	0.0192	5.561	0.0181
3	6.268	0.0026	5.995	0.0052	6.066	0.0057
4	6.352	0.0045	6.127	0.0024	6.187	0.0037
5	6.420	0.0271	6.295	0.0089	6.312	0.0890
6	6.606	0.0433	6.350	0.1631	6.338	0.0331
7	6.806	0.0150	6.416	0.0062	6.365	0.0090
8	7.004	0.2949	6.527	0.0326	6.580	0.0033

Table S2: Cartesian coordinates (in Å) for the evaluation of natural transition orbitals in Table 1 in the main text and excited state properties in Table S1.

Atom	x	y	z
S	-1.180470	-0.347480	0.276020
C	-0.011770	0.759220	-0.286790
N	0.743330	1.542220	-0.750180
O	-1.048470	-3.930380	-0.234780
H	-0.577570	-3.220280	-0.710790
H	-1.055770	-4.638680	-0.884990
O	4.495230	-0.678980	1.282120
H	4.217030	-0.556080	2.197610
H	5.401230	-1.005880	1.308310
O	-2.539670	2.295620	3.258010
H	-1.561670	2.332320	3.227310
H	-2.836070	3.071020	2.698210
O	3.364530	1.680820	0.407210

Table S2 – continued

Atom	x	y	z
H	2.481730	1.500520	-0.042780
H	3.694130	0.840320	0.779620
O	-2.408870	3.446820	-3.322790
H	-2.614670	3.229020	-2.391990
H	-1.547370	3.000320	-3.485690
O	-4.403170	-0.165180	-1.329580
H	-3.894070	-0.979180	-1.271980
H	-4.438470	-0.089480	-0.322780
O	0.216230	-0.634380	-3.566180
H	0.108730	0.330220	-3.614490
H	0.634230	-0.920180	-4.412980
O	0.109930	2.256720	-3.768390
H	0.657430	2.620820	-4.504290
H	0.780830	1.998020	-3.132480
O	-1.658670	-0.884180	3.113120
H	-1.635470	-0.621280	2.176510
H	-0.987870	-0.299780	3.487810
O	0.904530	-2.015280	1.988210
H	1.823630	-2.336180	1.797410
H	0.662130	-1.767280	1.114110
O	0.393430	-3.204780	-2.648290
H	0.192030	-2.261380	-2.759890
H	1.333530	-3.201080	-2.543390
O	2.975230	1.827820	-2.951390
H	2.308430	1.467320	-2.320690

Table S2 – continued

Atom	x	y	z
H	3.182430	1.132120	-3.619190
O	-3.175970	-2.589180	-1.088590
H	-3.503370	-3.111680	-1.770080
H	-2.555470	-3.166080	-0.705780
O	3.042630	-3.263680	-1.950090
H	2.782130	-2.826880	-1.111380
H	3.334930	-4.095080	-1.559580
O	-0.083070	3.429520	2.682210
H	-0.123770	4.007320	1.888010
H	0.102930	3.993420	3.428210
O	-3.993970	1.086920	1.415010
H	-3.151370	1.326020	1.848910
H	-4.013470	0.194920	1.811620
O	-2.983270	2.939220	-0.334180
H	-3.200070	3.736020	0.194720
H	-3.387670	2.186520	0.178010
O	3.082130	-2.894880	0.740520
H	3.392830	-3.848280	0.738110
H	3.852530	-2.301780	0.653920
O	3.479030	0.001120	3.528720
H	2.584930	0.371320	3.447720
H	3.386630	-0.663980	4.240520
O	0.930630	0.412620	3.004720
H	0.896730	0.953320	2.211620
H	0.835230	-0.483680	2.655310

Table S3: Mean excitation energies (eV) and oscillator strengths f obtained from all snapshots. Standard deviations are given in parentheses.

State	FDET(SCN^-)^{QM}		FDET(SCN^-+11H_2O)^{QM}		Ref[ADC(2)]	
	ϵ	f	ϵ	f	ϵ	f
1	5.454(0.195)	0.0262(0.0106)	5.479(0.214)	0.0276(0.0109)	5.502(0.223)	0.0255(0.0111)
2	5.609(0.187)	0.0260(0.0112)	5.612(0.210)	0.0353(0.0175)	5.629(0.217)	0.0339(0.0183)
3	6.272(0.158)	0.0108(0.0114)	6.073(0.155)	0.0088(0.0081)	6.150(0.175)	0.0103(0.0110)
4	6.393(0.150)	0.0131(0.0154)	6.206(0.163)	0.0205(0.0202)	6.269(0.181)	0.0211(0.0184)
5	6.515(0.145)	0.0130(0.0141)	6.332(0.166)	0.0239(0.0296)	6.394(0.181)	0.0256(0.0261)
6	6.737(0.173)	0.1323(0.1342)	6.507(0.174)	0.1382(0.1226)	6.505(0.175)	0.0748(0.0805)
7	6.887(0.195)	0.0648(0.0995)	6.664(0.191)	0.0278(0.0304)	6.618(0.180)	0.0261(0.0299)
8	7.116(0.163)	0.2109(0.2108)	6.828(0.185)	0.0892(0.1065)	6.756(0.154)	0.0320(0.0304)
